# Genetic differences associated with dopamine and serotonin release mediate fear-induced bradycardia in the human brain

**DOI:** 10.1038/s41398-024-02737-x

**Published:** 2024-01-15

**Authors:** Simone Battaglia, Claudio Nazzi, Julian F. Thayer

**Affiliations:** 1 Department of Psychology, Center for Studies and Research in Cognitive Neuroscience, University of Bologna, Bologna, 40127 Italy; 2 Department of Psychology, University of Torino, Torino, 10124 Italy; 3 Department of Psychological Science, 4334 Social and Behavioral Sciences Gateway, University of California, Irvine, CA 92697 USA; 4 Department of Psychology, The Ohio State University, Columbus, OH 43210 USA

**Keywords:** Clinical genetics, Molecular neuroscience, Predictive markers

## Abstract

Fear-induced bradycardia, a transient heartbeat deceleration following exposure to threat, is a physiological index observable in humans, especially in fear conditioning experiments. While gaining interest in recent years, it is still currently underemployed in neuroscientific research compared to more popular physiological indices. Besides its use in research, it could also constitute a valuable resource in a clinical psychiatry setting, as many disorders are also characterized by altered heart rate responses. However, differences in fear-induced bradycardia may also be subtended by genetic interindividual differences, thus suggesting precaution when recommending its use in the clinical setting. Here, we discussed the first endeavors that aimed at clarifying the genetic underpinnings of heart rate variations, which suggest that individual genetic differences have a role in defining the characteristics of heart rate responses. Given this, translating heart rate measurements in the clinical setting must be implemented with caution. Future endeavors in this field will aim at identifying these differences even further, thus allowing for more precise clinical interventions.

## Introduction

The use of fear conditioning in the neuroscience and psychiatry domains is beyond well established, thanks to its flexibility and the chances it provides to study multiple phenomena. In humans, it is used to explain the theoretical model behind the development of different psychiatric disorders and to investigate their symptoms, while also allowing to reveal the neurocircuitry behind dysfunctional fear learning processes [1]. Fear conditioning is based on the theoretical principle that a neutral stimulus, when repeatedly presented with a painful, threatening, or unpleasant consequence, becomes a conditioned stimulus (CS). This implies that, even when presented alone, it can elicit the same cascade of physiological and psychological changes that can be observed when being subjected to a highly threatening and direct consequence. In humans, these changes usually can be measured by a wide range of neuroscientific techniques, namely skin conductance resistance (SCR), fear-potentiated startle (FPS), and pupillary responses (PR), among others.

Notwithstanding, heart rate (HR) changes and the study of variability (HRV) are becoming more popular techniques, thanks to modern innovations overcoming data analysis challenges using physiology-based algorithms as well as machine learning approaches [2]. In comparison to the other techniques, HR allows us to accurately assess fear responses even when a large number of trials is required, as the magnitude of HR changes remains constant through time [3]. Accordingly, recent growing interest is related to a physiological index that has been identified in this theoretical framework and called ‘fear-induced bradycardia’, a rapid and transient heartbeat deceleration arising after exposure to a threatening stimulus [2–4] and error monitoring [5]. Despite the historical root of HR employment goes back over a century, fear-induced bradycardia is still employed as a secondary measure among other more popular alternatives, even though recent works gave way to tangible evolutions that highlighted the unique features of this biomarker, even in the psychiatric domain.

Notably, as fear conditioning in humans allows us to study both the normal processes as well as the aberrant mechanisms, its relevance extends beyond the realm of mere neuroscientific research and reaches the psychiatric clinical setting. Indeed, defective manifestations of fear conditioning may be a common feature of many psychiatric disorders [6–8]—a clearer understanding of such peculiarities could be key in revealing possible uses of fear-induced bradycardia as a biomarker for even more precise and timely diagnoses. As 20–60% of psychiatric patients respond poorly to treatments, with grave economic and societal burdens on the healthcare system, more up-to-date treatments resulting from these advances will help in reducing direct and indirect costs originating from psychiatric treatments [9].

Nevertheless, may this hopeful prospect be too optimistic? Fear-induced bradycardia is a solid enough phenomenon in the context of traditional, non-clinical research, but the possibility to employ it as a functional biomarker in clinical applications must be pondered. Importantly, it has been found that neurotransmitter-related genetic variations influence the manifestation of HR changes in fear conditioning [10, 11]. Therefore, it is reasonable to consider that individual genetic characteristics may have an impact on the expression of fear responses too. In this manuscript, we aim at discussing the evidence about genetic differences, and their impact on the serotoninergic and dopaminergic systems and, in turn, on the expression of fear-induced bradycardia in humans.

## Genetic-mediated serotonin and dopamine systems differences in fear-induced bradycardia

**Fig. 1:**
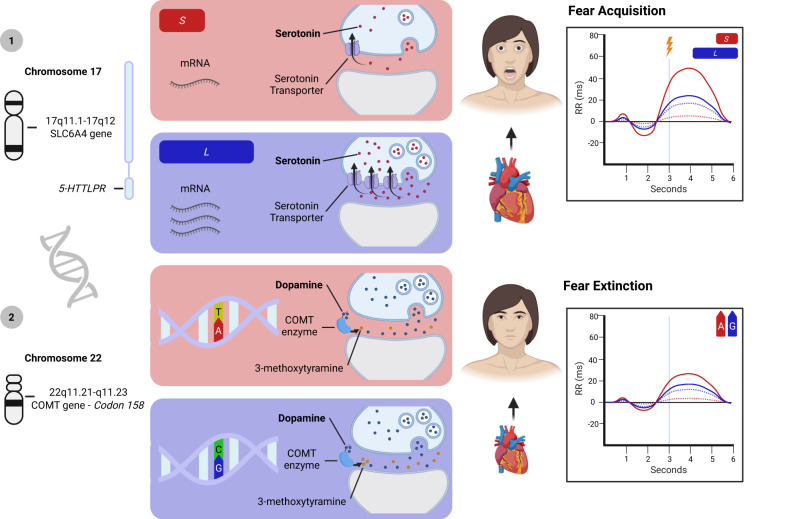
Graphical representation of genetic differences influencing serotonin and dopamine and their repercussions on fear-induced bradycardia. (**1**) The serotonin transporter linked promoter region (5HTTLPR) of the solute carrier family 6 member 4 (SLC6A4) gene is polymorphic. The short (*S*) version results in lower messenger ribonucleic acid (mRNA) transcription in human cell lines, leading to a lower amount of serotonin transported back to the presynaptic neuron. On the other hand, the long (*L*) allele is associated with higher mRNA transcription and greater serotonin reuptake. The results from Shipper and colleagues’ work (2019) suggest that *S* carriers show greater fear-induced bradycardia compared to *L* carriers. Moreover, only the *S* carriers showed a differential response between conditioned stimuli (CS) and neutral ones (continuous lines represent CSs, dotted lines represent neutral stimuli). (**2**) A single-nucleotide polymorphism on codon 158 of the catechol-O-methyltransferase gene (COMT) leads to differences in the activity of the COMT enzyme, responsible for breaking down dopamine in 3-methoxytyramine. The A nucleotide variant is associated with a valine (Val) to methionine (Met) amino acid change, which results in lower COMT activity and higher synaptic dopamine levels. Therefore, COMT activity is lowest in met homozygotes and highest in val homozygotes. Evidence from Panitz and colleagues’ study (2018) suggests that only val homozygotes showed good extinction retention, as reflected by lower differential heart rate (HR) between the CSs presented during extinction (continuous lines) compared to the CSs that were not presented during that phase (dotted lines).

Serotonin (5-HT) is a neurotransmitter in the nervous system, with a widespread role in physiology that is also involved in numerous psychiatric conditions [12]. 5-HT is critically implicated in a series of brain functions, such as mood, sleep, and appetite regulation [13]. As increased synaptic availability of 5-HT in the central nervous system is linked to mood improvements and a reduction of anxiety [14], it forms the foundations of different well-known anti-depressants, which act by preventing the removal of 5-HT from the synaptic space [15]. Accordingly, it has been reported that a reduced expression of the serotonin transporter (5-HTT) is known to play a significant role in the development of stress-related psychopathology [16]. To test its impact on fear-induced bradycardia, Schipper and colleagues [10] recruited a total of 104 participants, which were split into two groups based on their genetic variant of the 5-HTT promoter (5-HTTLPR). One group consisted of short (*S*) allelic variant carriers, which is associated with reduced transcription and function of 5-HTT when compared with the long (*L*) allele [17], while the other group consisted of *L* homozygotes only. All participants underwent a fear conditioning experiment, consisting of an acquisition phase only during which one of two visual stimuli was paired with a shock. The experiment was carried out while having participants undergo functional magnetic resonance, in order to also gather neuroimaging data. Results showed that the *5-HTTLPR* genotype modulates fear-induced bradycardia expression since *S* carriers showed stronger heart rate slowing compared to *L* homozygotes. Moreover, only *S* carriers showed differential HR response when comparing CSs to neutral stimuli, and neuroimaging data revealed a reduced medial prefrontal cortex activation and an increased connectivity between the amygdala and the periaqueductal gray. Crucially, results from this study reveal that genetic differences have a selective and direct impact on the manifestation of fear-induced bradycardia. In particular, this suggests that a lower amount of serotonin transporter can lead to an increased expression of fear responses in humans. However, it is important to highlight that when considering the main effect of stimulus type—irrespective of groups—on HR responses, CS presentation fosters a greater deceleration compared to neutral stimuli. This evidence on genetic variations could suggest that when both *S* carriers and *L* homozygotes are part of a single experimental group, fear-induced bradycardia may still be observable, possibly because of the larger impact on such measure that *S* carriers bring (Fig. 1).

In addition to serotonin, dopamine also plays a major role in health, thanks to its influence on motor control, reward, and cognitive function [18], and in psychiatric disease [19]. It is implicated in the generation of movement [20], and different addictive drugs act by either increasing dopamine levels or blocking its reuptake [21]. Crucially, its predominant role is the generation of wanting pleasurable rewards [22]. Indeed, differences in dopamine levels have been associated with different learning phenomena, including fear extinction [23], a process that allows to interfere with the recall mechanism behind the association between the CS and the threatening stimulus; usually, it can occur by repeatedly presenting the CS alone. Therefore, genetic differences may also have an impact on its expression as Panitz and colleagues investigated [11]. In particular, authors reported that *Val158Met* single-nucleotide polymorphism on the catechol-O-methyltransferase gene (COMT), which is responsible for individual differences in prefrontal dopamine, may be linked to individual differences in human fear extinction. Authors recruited 87 participants, divided into three groups based on genotype distribution (which could be val/val, val/met, met/met). They underwent a fear conditioning paradigm consisting of a habituation, acquisition, and extinction phase on the first day and a recall phase on the following day. The stimuli used were four pictures of faces, two of which were associated with a white noise burst serving as US while the other two were never paired with the US. During fear extinction, only one CS and one neutral stimulus were presented, so that in the recall phase, during which all stimuli were presented again, authors compared physiological responses to extinguished and non-extinguished CSs, as well as to the neutral stimulus that was presented during extinction the one that was not. Critically, results showed that during the recall phase on the following day, while fear-induced bradycardia was greater for CSs in general, only the val/val group showed good extinction retention, as reflected by lower differential HR between the CSs presented during extinction compared to the CSs that were not presented during that phase. The evidence carried by this study demonstrated that the genotype of Val homozygotes expresses better retention of previously learned extinction, suggesting that genetic differences can have an impact on fear-induced bradycardia expression (Fig. 1).

## Discussion

The present manuscript discussed the evidence regarding the influence of genetic differences in HR responses during human fear conditioning experiments. Studies showed that the genetic characteristics of individuals, particularly those related to neurotransmitter differences, have an impact on fear-induced bradycardia. Specifically, a lower amount of serotonin transporter is linked to higher differential responses between neutral and conditioned stimuli [10], while lower dopamine levels have a positive impact on correctly recalling fear extinction on the day following conditioning [11]. By gathering the evidence, it is possible to provide a theoretical overview that encapsulates the effort brought forward within this field.

However, data collected in humans on this focused domain is limited and circumscribed so far. Nonetheless, the insights provided are vital in bringing forward more in-depth knowledge regarding novel biomarkers and their possible uses in clinical psychiatry. This possibility must come with some caution, as to avoid coming to improper conclusions, given the low number of studies. Fear-induced bradycardia might be a valuable biomarker, able to detect physiological changes in psychiatric populations when more traditional techniques, like SCR or FPS, apparently reveal no differences [24, 25]. Furthermore, there is evidence that fear-induced bradycardia manifestations are altered in a wide array of psychiatric populations [26, 27]. Finally, its application in the clinical setting may prove to be highly useful, but the two studies analyzed here suggest moving with caution. The reasoning behind this precautionary statement is subtended by the idea that if changes to fear-induced bradycardia are evident in the case of genetic differences, then those differences may be at the core of HR response aberrations, and not psychiatric disorders. Future investigations will provide further foundations upon which to build novel interventions, which will help in monitoring treatment efficacy and follow-up evaluations.

It is well known that genetic factors have an impact on the development of psychiatric disorders [28]. There is a high level of genetic heritability for different disorders [29], and there is evidence that some genetic risk factors are shared among different psychiatric illnesses [30]. Moreover, individual variations in the different phases of the fear conditioning process are linked to genetic differences [31]. In line with this corroborated evidence, the hypothesis that aberrant fear conditioning is a common cause behind the development of different psychiatric disorders [6] is also well established. This suggests a link between the influence of genetic differences on fear memories and on the development of psychiatric disorders. In a clinical setting, however, this could be a double-edged sword. The implementation of fear-induced bradycardia as a biomarker for psychiatry is highly valuable, as the presence or absence of such a phenomenon in specific conditions could be a manifestation of specific disorders. Yet, as shown by the studies discussed here, the genotype distribution of patients may already have an influence over the expression of fear-induced bradycardia, potentially lowering the accuracy of this methodology. Nonetheless, future investigations will be needed to aim to more specifically define the influence of genetic differences on fear-induced bradycardia, thus elevating it as a potential tool in the diagnosis of psychiatric disorders.

## Conclusion

In conclusion, the evidence discussed in this manuscript should not call for a dismissal of new methodologies and protocols in the neuro-psychiatric clinical setting, but rather an emphasis on the possible pitfalls that may come from overly optimistic hypotheses. However, given the evidence that psychiatric disorders have aberrant HR responses [26, 27], the possibility of using those in the clinical setting needs to be explored. In the near future, new techniques, advanced real-time analysis algorithms, machine learning, as well as physiological biomarkers may streamline the mental healthcare process, also alleviating the social burden and economic pressures commonly associated with psychiatric disorders. This can be done by focusing on genetic differences, how they impact the serotoninergic and dopaminergic systems, and, in turn, on the expression of fear-induced bradycardia in humans.
